# Inhibition of HDAC1 and DNMT1 Modulate RGS10 Expression and Decrease Ovarian Cancer Chemoresistance

**DOI:** 10.1371/journal.pone.0087455

**Published:** 2014-01-27

**Authors:** Ercan Cacan, Mourad W. Ali, Nathaniel H. Boyd, Shelley B. Hooks, Susanna F. Greer

**Affiliations:** 1 Division of Cellular Biology and Immunology, Center for Inflammation, Immunity and Infection, Department of Biology, Georgia State University, Atlanta, Georgia, United States of America; 2 Department of Pharmaceutical and Biomedical Sciences, University of Georgia, Athens, Georgia, United States of America; Emory University, United States of America

## Abstract

RGS10 is an important regulator of cell survival and chemoresistance in ovarian cancer. We recently showed that RGS10 transcript expression is suppressed during acquired chemoresistance in ovarian cancer. The suppression of RGS10 is due to DNA hypermethylation and histone deacetylation, two important mechanisms that contribute to silencing of tumor suppressor genes during cancer progression. Here, we fully investigate the molecular mechanisms of epigenetic silencing of RGS10 expression in chemoresistant A2780-AD ovarian cancer cells. We identify two important epigenetic regulators, HDAC1 and DNMT1, that exhibit aberrant association with RGS10 promoters in chemoresistant ovarian cancer cells. Knockdown of HDAC1 or DNMT1 expression, and pharmacological inhibition of DNMT or HDAC enzymatic activity, significantly increases RGS10 expression and cisplatin-mediated cell death. Finally, DNMT1 knock down also decreases HDAC1 binding to the RGS10 promoter in chemoresistant cells, suggesting HDAC1 recruitment to RGS10 promoters requires DNMT1 activity. Our results suggest that HDAC1 and DNMT1 contribute to the suppression of RGS10 during acquired chemoresistance and support inhibition of HDAC1 and DNMT1 as an adjuvant therapeutic approach to overcome ovarian cancer chemoresistance.

## Introduction

Ovarian cancer is one of the deadliest gynecological cancers, with a 60% mortality rate in patients and a 5-year survival rate of lower than 30% in advanced stage disease [1]. The high mortality rate is due in large part to the development of resistance to chemotherapeutic drugs [2], [3]. Thus, understanding the molecular and genetic mechanisms that drive the development of acquired chemoresistance will enable us to improve current therapeutic agents for ovarian cancer treatment. G-protein coupled receptors (GPCRs) initiate multiple oncogenic signaling pathways in cancer cells by activating their associated G-proteins [4], [5]. Activation of GPCRs by growth factors such as Lysophosphatidic acid (LPA) triggers survival signaling pathways that drive resistance to chemotherapeutic drugs such as cisplatin and taxane [6]. GPCR activation of G-proteins is opposed by the activity of regulator of G-protein signaling (RGS) proteins. RGS proteins inhibit G-protein signaling pathways by directly binding to the activated Gα subunit of G-proteins to accelerate hydrolysis of GTP into GDP, which returns G-proteins to an inactive state [7]–[10]. Relevant to our studies, recent reports indicate that RGS proteins inhibit breast, lung, prostate, and ovarian cancer cell growth through inhibition of GPCRs signaling pathways [2], [11]–[15].

RGS10 is among the smallest of the RGS proteins and is highly expressed in a broad range of cell types [16]–[19]. RGS10 is an important regulator of cell survival and chemoresistance [2], and RGS10 transcript expression is significantly suppressed in multiple ovarian cancer cell lines [15]. Thus, the suppression of RGS10 proteins may contribute to chemoresistance by amplifying GPCR-mediated cell growth and survival signaling pathways. We have recently shown that suppression of RGS10 is due in part to DNA hypermethylation and to histone deacetylation, two important gene-silencing mechanisms which contribute to the progression of many cancers. DNA methylation is maintained by DNA methyl transferases (DNMTs) [20] and histone deacetylation is maintained by histone deacetylases (HDACs) [21]. Often, these two enzymes coordinately suppress transcriptional activity of genes [22], [23]. Fuks *et al.* have reported that DNMT1 is associated with histone deacetylase activity and has the ability to bind HDAC1 [24]. However, the molecular mechanisms by which DNA hypermethylation and histone deacetylation suppress RGS10 and the contribution of these enzymes to acquired chemoresistance remains unknown.

We investigate here the molecular mechanisms of epigenetic regulation of RGS10 expression in ovarian cancer cells and focus on chemosensitive parental A2780 cells and their derivative cell line, chemoresistant A2780-AD. We identify two important epigenetic regulators, HDAC1 and DNMT1, which are highly associated with the RGS10 promoter in chemoresistant ovarian cancer cells. HDAC1 and DNMT1 knock down significantly increases RGS10 expression and cisplatin-stimulated cell death. Our results suggest that HDAC1 and DNMT1 contribute to the suppression of RGS10 during acquired chemoresistance and support growing evidence that inhibition of HDAC1/DNMT1 represent novel therapeutic approaches to overcoming ovarian cancer chemoresistance.

## Materials and Methods

### Cell lines and reagents

The chemosensitive A2780 parental cell line and their derivative chemoresistant A2780-AD cells (derived as described [25]) were generously provided by Dr. Bob Brown, Imperial College London. These cells were maintained in RPMI 1640 medium (Mediatech Inc.) supplemented with 10% FBS and 5 mM L-glutamine. Chemoresistant cells were further maintained in 3 µM cisplatin. All cells were grown in 5 mM penicillin-streptomycin at 37°C with 5% CO_2_. OV2008 and C13 cells (derived as described [26], [27]) were generously provided by Dr. Patricia Kruk, University of South Florida.

5-Aza-2′-deoxycytidine (5-Aza-dC), Trichostatin A (TSA), and cisplatin were purchased from Sigma-Aldrich (St. Louis, MO). Antibodies recognizing RGS10, HDAC1, goat-anti-rat IgG-HRP (Horseradish peroxidase), and HRP conjugated rabbit antibodies were obtained from Santa Cruz (Santa Cruz, CA). Antibodies recognizing DNMT1 were obtained from Abcam (Cambridge, MA). HRP conjugated mouse antibodies were purchased from Promega (Madison, WI). Antibodies recognizing β-Actin were obtained from Cell Signaling (Beverly, MA).

### siRNA constructs and transient transfection

Short interfering RNA (siRNA) pre-designed for HDAC1 (Qiagen), RGS10 and DNMT1 (Santa Cruz) were used to knock down expression of HDAC1, RGS10 or DNMT1. Scrambled All Star Control siRNA (Qiagen) was used as a control. A2780-AD cells were transfected with 10 nM of HDAC1 or DNMT1 specific siRNA or All Star scrambled control siRNA using HiPerfect transfection reagent (Qiagen) according to the manufacturer's instruction. Following indicated incubation time, cells were harvested and analyzed in western blot, RNA expression, or chromatin immunoprecipitation experiments.

### RNA expression and quantitative real-time PCR

mRNA was isolated using Qiazol RNA extraction reagent (Qiagen) as described in the manufacturer's protocol. Briefly, cells were lysed in Qiazol and agitated on a 3D rotator for 5 minutes. 200 µl of chloroform was added and was incubated for three minutes at room temperature. Samples were centrifugated and the aqueous phase (400 µl) was transferred to an eppendorf tube. 500 µl of isopropanol was added and was incubated for 10 minutes at room temperature. Following centrifugation, pellets were washed with 1 mL of cold 75% ethanol, centrifuged and resuspended in 50 µl of RNAse free water. RNA was quantified and cDNA was generated from 1 µg of total extracted RNA using an Omniscript Reverse Transcription Kit (Qiagen). Following cDNA synthesis, quantitative real-time polymerase chain reaction was performed using TaqMan Universal PCR Master Mix (Roche) and specific primers and probes targeting RGS10 or GAPDH coding regions. Transcript expression was assessed using an ABI prism 7900HT Real-Time PCR System (Applied Biosystems). Reactions were normalized against GAPDH expression and calculations were performed using standard curves generated. Primers used were: RGS10 Forward: 5′-GAC CCA AGA AGG CGT GAA AAG A-3′, RGS10 Reverse: 5′-GCT GGA CAG AAA GGT CAT GTA GA-3′, RGS10 probe: 5′-AGA TAA GAC GCA GAT GCA GGA AAA GGC-3′, GAPDH Forward: 5′-GGA AGC TCA CTG GCA TGG C-3′, GAPDH Reverse: 5′-TAG ACG GCA GGT CAG GTC CA-3′ and GAPDH probe: 5′- CCC CAC TGC CAA CGT GTC AGT G-3′.

To determine the effect of 5-Aza-dC and TSA exposure on RGS10 transcript expression, 1×10^6^ A2780-AD cells were plated per 10 cm^2^ tissue culture plate and were incubated overnight. Cells were treated with 20 µM 5-Aza-dC or with 500 ng TSA dissolved in DMSO. TSA treated cells were incubated for 48 hours and 5-Aza-dC treated cells were incubated for three, five, or seven days, with media aspirated and replaced daily. RNA isolation and DNA synthesis were performed as described above.

### Chromatin immunoprecipitation (ChIP) assay

ChIP assays were performed as previously described [15]. Briefly, cells were plated at a density of 2×10^6^ in 10 cm^2^ plates. Three million cells were crosslinked with 1% formaldehyde for eight minutes at room temperature. Crosslinking reactions were stopped by the addition of 0.125 M glycine. Cell nuclei were isolated and were concentrated by lysis in SDS lysis buffer (1% SDS, 10 mM EDTA, 50 mM Tris pH 8.0) plus protease inhibitors for 30 minutes on ice followed by flash freezing in liquid nitrogen. Nuclei were sonicated using a Bioruptor water bath sonicator (Diagenode) to generate an average of 500 bp of sheared DNA which was confirmed by agarose gel electrophoresis. Sonicated lysates were precleared with salmon-sperm/agarose beads (Millipore), 5% of the total lysate was stored as input for normalization. Half of the remaining lysate was immunoprecipitated with 5 µg of indicated antibody overnight at 4°C and the other half of the lysate was immunoprecipitated with a control antibody. Following an additional two hour immunoprecipitation with salmon-sperm coated agarose beads, all samples were washed with each of the following buffers: low salt buffer (0.1% SDS, 1% Triton X-100, 2 mM EDTA, 20 mM Tris pH 8.0, 150 mM NaCl), high salt buffer (0.1% SDS, 1% Triton X-100, 2 mM EDTA, 20 mM Tris pH 8.0, 500 mM NaCl), LiCl (0.25 M LiCl, 1% NP40, 1% DOC, 1 mM EDTA, 10 mM Tris pH 8.0), and 1×TE (Tris-EDTA); DNA was then eluted with SDS elution buffer (1% SDS, 0.1 M NaHCO3). Following elution, cross-links were reversed overnight with 5 M NaCl at 65°C and the immunoprecipitated DNA was isolated using phenol:chloroform:isopropanol mix (Invitrogen) as per the manufacturer's instructions. Isolated DNA was quantified by real time PCR on an ABI prism 7900 HT (Applied Biosystems, Foster City, CA) using specific primers and probes targeting RGS10 and GAPDH promoters region. Values generated from real time PCR reactions were calculated based on standard curves generated, were run in triplicate reactions, and were analyzed using the SDS 2.0 program (Applied Biosystems).

### Chromatin immunoprecipitation assay in siRNA treated cells

Chemoresistant A2780-AD cells were plated at a density of 1.2×10^6^ cells per 10 cm^2^ tissue culture plates. Cells were treated with siRNA constructs as described above and were incubated for 72 hours. Cells were harvested and 1/10 of the cell volume was removed for analysis of knockdown efficiency by western blot analysis. ChIP assays were carried out as described above on the remaining harvested cells.

### RNA expression in siRNA treated cells

Cells were plated at a density of 1×10^6^ cells per 10 cm^2^ tissue culture plate and were incubated overnight. The cells were then transfected with the indicated siRNA construct as described above and incubated for 72 hours, and an RNA extraction was performed as described above.

### Apoptosis assay

Apoptosis of A2780-AD cells was assessed using the Annexin V: PE Apoptosis Detection Kit I (BD Pharmingen). 1×10^6^ A2780-AD cells were plated on a 10 cm^2^ tissue culture plate and were incubated for 24 hours at 37°C. The cells were treated with HDAC1 siRNA or with control siRNA using the HiPerfect transfection reagent. Following 48 hours incubation, 50 µM of cisplatin was added to both HDAC1 siRNA and control siRNA treated cells and the cells were incubated for an additional 48 hours. A2780-AD cells were briefly trypsinized and were harvested. A fraction of the cell volume was removed for western blot analysis and the remaining fraction of cells was washed with cold PBS twice and was resuspended in Annexin V binding buffer at a concentration of 1×10^6^ cells/ml. The cells were then transferred to 5 mL culture tubes containing 5 µl of Annexin V-PE and/or 5 µl of 7-Aminoactinomycin D (7-AAD). The samples were gently mixed and were incubated for 20 minutes at room temperature. Following the addition of 400 µl of Annexin V binding buffer to each tube, samples were analyzed and quantified by flow cytometry and resulting data were analyzed using FlowJo software.

## Results

### Histone acetylation is suppressed at RGS10 promoters in ovarian cancer cells

**Figure 1:**
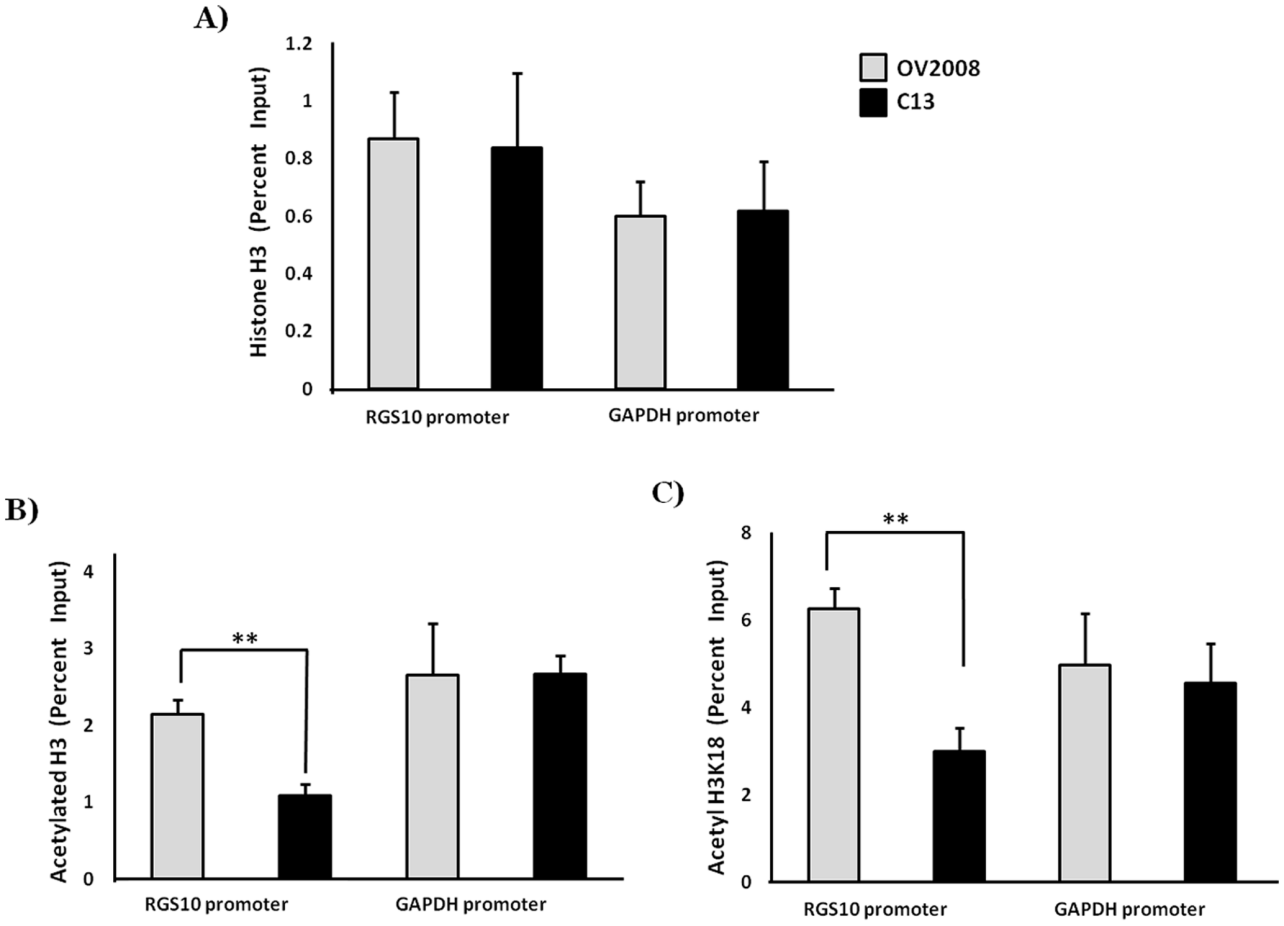
Histone H3 and acetylation levels at RGS10 promoters in chemoresistant C13 cells and parental OV2008 cells. ChIP assays were carried out in OV2008 parental cells and drug resistant C13 cells. Lysates were immunoprecipitated with control, anti-histone H3, anti-acetyl histone H3, or anti-acetyl H3K18 antibodies. Associated DNA was isolated and analyzed via real time PCR using primers spanning the RGS10 and GAPDH promoters. Real-time PCR values were normalized to the total amount of promoter DNA added (input). Input values represent 5% of the total cell lysate. **p<0.005. **A**) Global histone H3 levels associated with RGS10 and GAPDH promoters. **B**) Global levels of histone H3 acetylation associated with RGS10 and GAPDH promoters. **C**) Levels of histone H3 acetylated at lysine 18 associated with RGS10 and GAPDH promoters.

We have recently shown that levels of acetylated histone H3 histone are significantly decreased at the RGS10-1 promoter in the A2780-AD cell model of ovarian cancer chemoresistance [15]. To confirm these findings in additional cell lines, chromatin immunoprecipitation (ChIP) assays were carried out in the chemosensitive ovarian cancer cell line OV2008 and in chemoresistant C13 daughter cells. While total levels of histone H3 are similar at both RGS10 and GAPDH promoters in chemosensitive and chemoresistant cells (Fig. 1A), levels of acetylated histone H3 are significantly lower at RGS10 promoters in the chemoresistant C13 ovarian cancer cells as compared to chemosensitive OV2008 cells (Fig. 1B).

Reductions in H3 lysine 18 acetylation (H3K18Ac) have been associated with aggressive cancer phenotypes and with poor patient prognosis [28], [29]. We next performed ChIP assays to determine if loss of H3K18 contributes to the loss of global histone acetylation at RGS10 promoters in chemoresistant C13 cells. A significant decrease in H3K18 acetylation at RGS10 promoters was observed in chemoresistant C13 cells, while H3K18 acetylation at the GAPDH promoters in OV2008 and C13 cells remained unchanged (Fig. 1C). Together these data suggest the loss of acetylation at RGS10 promoters contributes to the loss of RGS10 expression in two independent cell models of chemoresistant ovarian cancer.

### HDAC1 and DNMT1 suppress RGS10 expression in chemoresistant ovarian cancer cells

**Figure 2:**
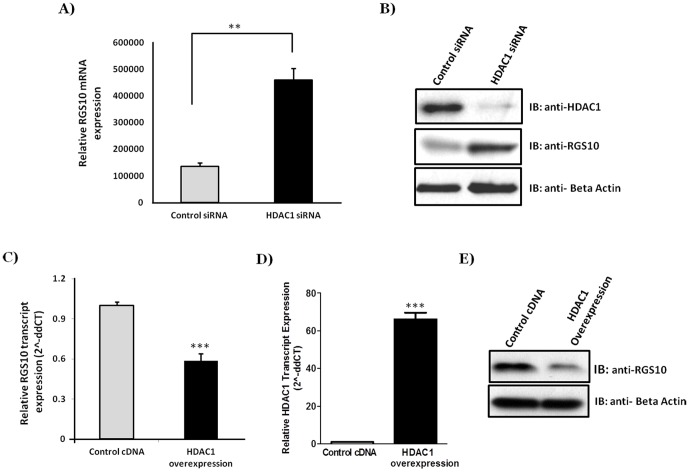
HDAC1 knockdown significantly increases RGS10 expression while HDAC1 overexpression decreases RGS10 expression in chemoresistant ovarian cancer cells. **A)** Knockdown of HDAC1 increases RGS10 mRNA transcription. A2780-AD cells were transfected with HDAC1 siRNA or control siRNA and incubated for 72 hours. RNA was extracted and cDNA was generated by using a reverse primer targeting for RGS10 or GAPDH coding region. Data was quantified using qPCR with primers and probes specific for RGS10 and GAPDH coding regions. Graphed data shows the average of three independent experiments, with error bars denoting standard error of the mean (SEM). Significance was calculated with a Student's t test **p<0.005. **B)** Western blot analysis demonstrating efficiency of HDAC1 knockdown and RGS10 protein expression following HDAC1 knock down with Beta-Actin controls. **C–D)** HDAC1 overexpression decreases RGS10 expression in chemoresistant cells. A2780/AD cells were plated in 24-well plate and allowed to attach overnight. Cells were transfected with 500 ng HDAC1 or empty vector using FuGene 6 reagent (Promega) according to the manufacturer's protocol. Following 48 hours incubation, cells were harvested in TRIzol (Invitrogen) and the expression of RGS10 and HDAC1 genes was assessed using RT-PCR as described, and normalized to actin gene expression. Values represent mean ± SEM of four independent experiments ***p<0.0005. **E)** Western blot analysis demonstrating RGS10 protein expression following HDAC1 overexpression with Beta-Actin controls.

We previously demonstrated that HDAC1 proteins bind with significantly increased frequency to the RGS10 promoter in chemoresistant A2780-AD cells compared to parental chemosensitive A2780 cells [15]. To investigate molecular roles for HDAC1 in regulating RGS10 expression, a siRNA duplex was utilized to specifically knock down endogenous HDAC1 expression in A2780-AD cells. siRNA-mediated knockdown of HDAC1 resulted in a more than 3-fold increase in endogenous RGS10 transcript expression as compared to control siRNA (Fig. 2A), suggesting that HDAC1 plays a critical role in regulating RGS10 transcription. Western blot analysis confirmed HDAC1 knock down increased RGS10 protein expression (Fig.2B). In a similar experiment, A2780-AD cells were transfected with HDAC1 to determine the effects of ectopic expression of HDAC1. Overexpression of HDAC1 dramatically reduced RGS10 expression in chemoresistant A2780-AD cells (Fig. 2C–E). Together, these data indicate that HDAC1 accumulation at RGS10 promoters likely contributes to suppression of RGS10 in chemoresistant ovarian cancer cells.

**Figure 3:**
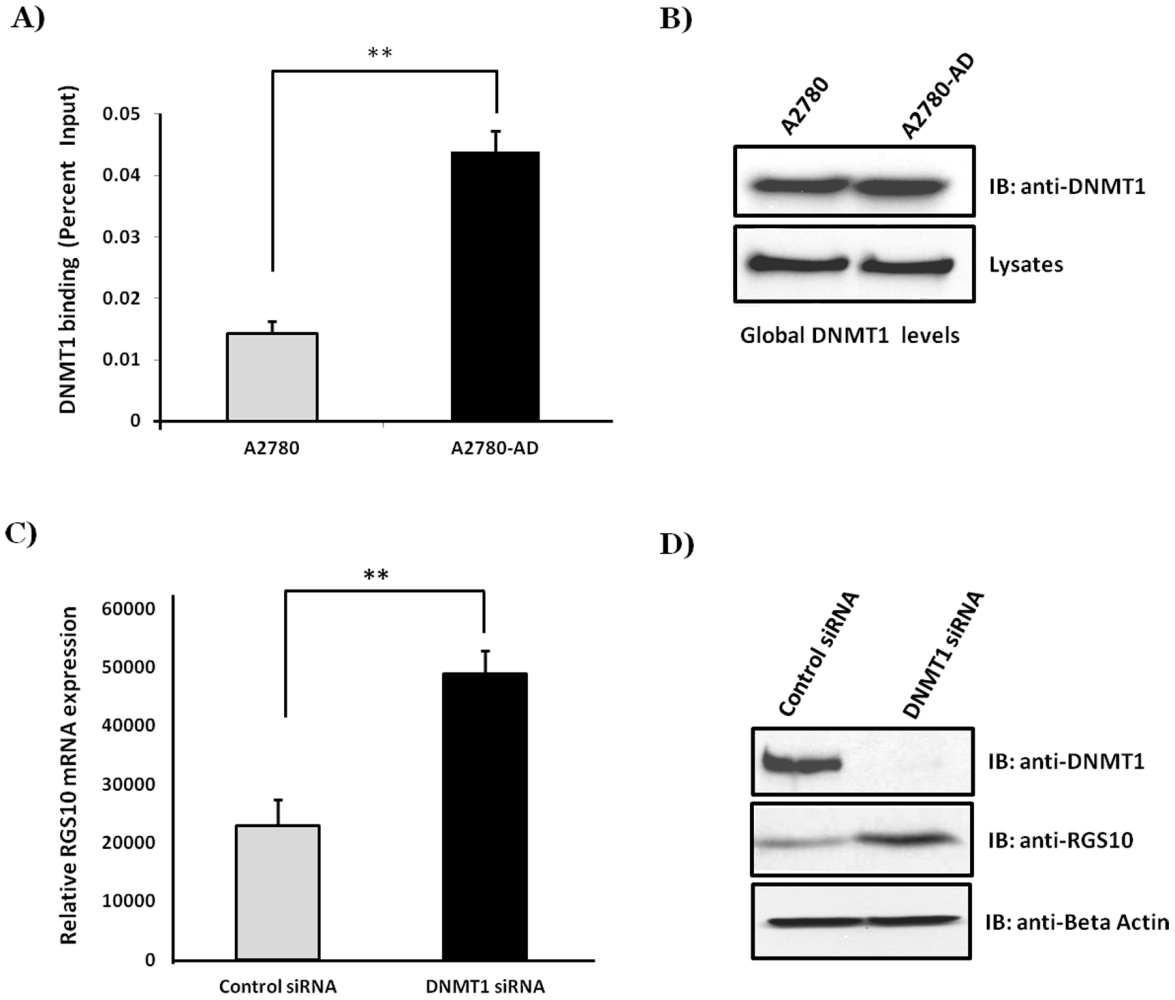
DNMT1 binding is increased at RGS10 promoter and DNMT1 knock down enhances the RGS10 transcript expression in chemoresistant ovarian cancer cells. **A)** Levels of DNMT1 associated with RGS10 promoters in A2780 and A2780-AD ovarian cancer cells. ChIP assays were carried out in A2780 parental and their derivative resistant A2780-AD cells. Lysates were immunoprecipitated with control or anti-DNMT1 antibody. Associated DNA was isolated and analyzed via real time PCR using specific primers and probes spanning the RGS10 and GAPDH promoters. Real-time PCR values were normalized to the total amount of promoter DNA added (input). Values were normalized to GAPDH and represent mean ± SEM of three independent experiments **p<0.005. **B)** Western blot analysis of global DNMT1 levels in A2780 and A2780-AD cells. Cells were harvested and lysates were used for immunoprecipitation. Antibody-conjugated agarose beads (IP) for DNMT1 were incubated with rotation overnight. Beads were washed, eluted and subjected to western blot with respective antibodies. **C)** A2780-AD cells were transfected with DNMT1 siRNA or with control siRNA and were incubated for 72 hours. RNA was extracted and cDNA was generated by using reverse primers targeting RGS10 and GAPDH coding regions. Data generated was quantified using qRT-PCR with primers and probes specific for the RGS10 coding region. Values represent mean ± SEM of four independent experiments **p<0.005. **D)** Western blot analysis for efficiency of knocking down DNMT1 and for RGS10 protein expression following DNMT1 knock down.

The RGS10-1 promoter contains a high concentration of CpG dinucleotides making it a potential target for DNMT maintenance methylation during ovarian cancer progression. We first determined by western blot analysis that DNMT1 expression levels are similar in both A2780 and A2780-AD cell lines (Fig. 3B). To further explore the relevance of DNMT1 in the specific suppression of RGS10 expression, ChIP assays were carried out in A2780 parental and their derivative resistant A2780-AD cells. Lysates were immunoprecipitated with control or anti-DNMT1 antibody and associated DNA was analyzed via quantitative real-time PCR (qRT-PCR) using specific primers and probes spanning the RGS10 and GAPDH promoters. In contrast to total protein abundance, ChIP assays reveal that binding of DNMT1 is significantly increased at the RGS10 promoter in chemoresistant cells as compared to chemosensitive ovarian cancer cells (Fig. 3A). Together these data indicate that accumulation of DNMT1 at the RGS10 promoter likely contributes to suppression of RGS10 during ovarian cancer chemoresistance.

Accumulation of DNMT1 at the RGS10 promoter led us to determine if that accumulation affects RGS10 transcript expression level in chemoresistant cells. For this purpose, A2780-AD cells were transfected with DNMT1 siRNA or control siRNA. RNA was extracted and generated cDNA was quantified using qRT-PCR with primers and probes specific for the RGS10 coding region and normalized to housekeeping gene GAPDH expression. The data reveals that knocking down DNMT1 significantly increases endogenous RGS10 transcript expression (Fig. 3C) and protein expression (Fig. 3D) in A2780-AD cells. This increase suggests that DNMT1 functions with HDAC1 to regulate the suppression of RGS10 transcription in chemoresistant A2780-AD cells.

### Inhibition of HDAC and DNMT activity enhances RGS10 expression and decreases ovarian cancer cell viability

**Figure 4:**
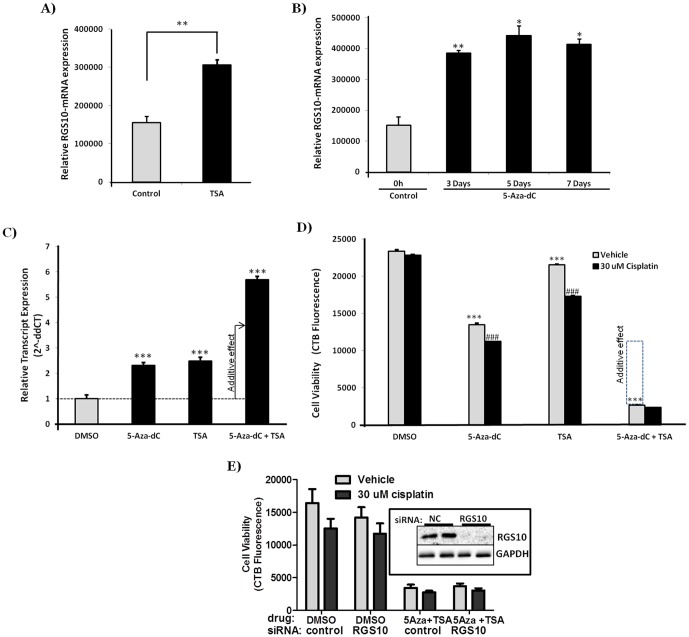
Effects of HDAC inhibitor trichostatin A (TSA) and DNMT inhibitor 5-Aza-2′-deoxycytidine (5-Aza-dC) on RGS10 transcript expression and cell viabilities. Total RNA was isolated from untreated control cells and TSA or 5-Aza-dC treated cells. The relative expression of RGS10 mRNA was quantified by qRT-PCR and normalized to GAPDH transcript expression * p<0.05; ** p<0.005. **A**) HDAC inhibition increases RGS10 expression in chemoresistant A2780-AD cells. The cells were plated in a 10 cm^2^ plate and incubated for 24 hours. The following day, cells were treated with 500 nM TSA and were incubated for an additional 48 hours. **B**) DNMT inhibitor 5-Aza-dC enhances RGS10 expression in chemoresistant cells. Two million A2780-AD cells were seeded in 10 cm^2^ plates and were incubated for 24 hours. The following day, cells were treated with 20 µM 5-Aza-dC. Media and drug were refreshed every 24 hours. After indicated incubation time (3, 5, and 7 days), cells were harvested, mRNA was isolated, and cDNA was generated and quantified using qRT-PCR with specific primers and probe. **C**) A2780-AD cells were plated in 96-well plates and treated with 5 µM 5-Aza-dC for 5 days, 500 nM TSA for 36 hours, a combination of 5 µM 5-Aza-dC for 5 days and 500 nM TSA for the final 36 hours or DMSO. Gene expression was assessed using qRT-PCR as described, and normalized to RPL13A gene expression. The arrow indicates the expression level predicted by an additive effect of TSA and 5-Aza-dC. **D**) In a parallel experiment, A2780-AD cells were treated under the same conditions as 4C with or without 30 µM cisplatin for the final 12 hours. Cell survival was assessed using CellTiter-Blue fluorimetric viability assays. ***: p<0.001 comparing epigenetic drug to DMSO control in the absence of cisplatin. ###: p<0.001 comparing vehicle versus cisplatin treatment within epigenetic drug treatment groups. The dotted box indicates the cell viability predicted by an additive effect of TSA and 5-Aza-dC. **E**) A2780/AD cells (5000 cells/well) were plated in 96-well plate and transfected with negative control or RGS10 siRNA duplexes (Ambion Grand Island, NY) as per the manufacturer's protocol using Dharmafect1 transfection reagent (Dharmacon). Cells were dosed with a combination of 5 µM 5-Aza-dC for 3 days and 500 nM TSA for the last 36 h or DMSO. 30 µM cisplatin or vehicle was added for the last 12 h. Cell survival was assessed using CellTiter-Blue fluorimetric viability assays.

We next sought to determine if pharmacologic inhibitors of histone deacetylation and DNA methylation can alter the expression of RGS10 in chemoresistant ovarian cancer cells. The HDAC inhibitor TSA and DNMT inhibitor 5-Aza-dC were used to inhibit HDACs and DNMTs, respectively. A2780-AD cells were treated with 500 nM TSA and were incubated for 2 days or were treated with 20 µM 5-Aza-dC and incubated for 3, 5 and 7 days. Total RNA was isolated from untreated control cells, TSA, and 5-Aza-dC treated cells. The relative expression of RGS10 transcript expression was quantified by qRT-PCR and was normalized to GAPDH transcript expression. Consistent with observations from HDAC1 and DNMT1 knock down experiments, TSA and 5-Aza-dC treatments both significantly enhanced RGS10 transcript expression in chemoresistant A2780-AD cells (Fig. 4A and 4B). To explore potential synergistic roles for HDAC1 and DNMT1 in regulating RGS10 expression, combination studies were performed using TSA and 5-Aza-dC in chemoresistant ovarian cancer cells. Again, TSA or 5-Aza-dC alone enhanced RGS10 expression in A2780-AD cells, and the combination of these two drugs results in a fold increase in RGS10 expression greater than the sum of the individual effect, suggesting a potential cooperative effect (Fig. 4C). To investigate cooperative roles for HDAC1 and DNMT1 in cell growth and chemoresistance, A2780-AD cells were treated with TSA and/or 5-Aza-dC in the presence of cisplatin and cell viability assays were performed. 5-Aza-dC alone reduced cell growth by approximately 40%, while TSA alone had a modest but significant effect on cell viability; however, the combination of TSA and 5-Aza-dC inhibited cell viability by 90%. As expected, A2780-AD cells were resistant to cisplatin toxicity, but either TSA or 5-Aza-dC partially re-sensitized the cells to cisplatin-mediated cytotoxicity (Fig. 4D). To determine if RGS10 upregulation by TSA/5-Aza-dC combined treatment could fully account for loss of cell viability, we attempted to rescue cell viability in the presence of 5-Aza-dC and TSA with RGS10 siRNA. Not surprisingly, knock-down of RGS10 alone did not rescue cell viability, consistent with the broad range of HDAC and DNMT target genes in cancer cells (Fig. 4E). Thus, RGS10 coordinately regulates cell viability and chemosensitivity with additional HDAC and DNMT target genes.

### Knocking down HDAC1 enhances cisplatin-stimulated apoptosis in chemoresistant cells

**Figure 5:**
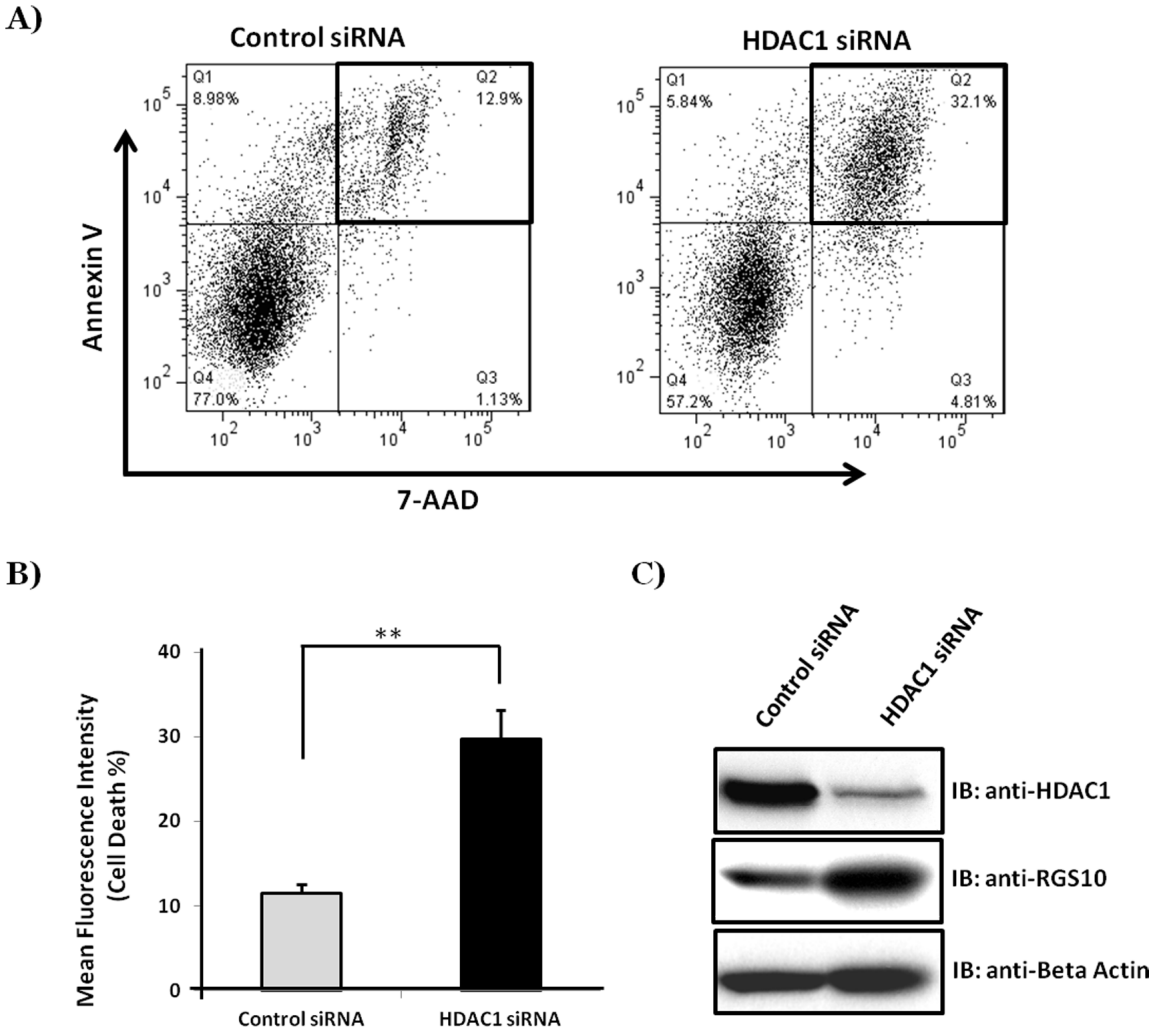
HDAC1 knockdown increases cisplatin stimulated apoptosis in chemoresistant cells. A2780-AD cells were treated with HDAC1 siRNA or control siRNA and were incubated for 48 hours. Following incubation, cells were treated with 50 µM cisplatin and incubated for addition 48 hours in RPMI 1640 media. An Annexin V: PE Apoptosis Detection Kit I (BD Pharmingen) was used for staining; results were quantified by Flow cytometry analysis and were analyzed using FlowJo software. Viable cells were negative for both annexin V-PE and 7-AAD; early apoptotic cells were positive for annexin V-PE and negative for 7-AAD, whereas late apoptotic dead cells were positive for both annexin V-PE and 7-AAD labeling. **A)** The relative increase of apoptotic cells in HDAC1- siRNA transfected A2780-AD cells which incorporated the Annexin V-PE and 7-AAD stains. **B)** Graph represent average of three independent experiments, with error bars denoting SEM *p<0.05. **C)** Western blot analysis represents efficiency of HDAC1 knockdown and RGS10 protein expression following HDAC1 knock down.

Our previous work suggests that suppression of RGS10 expression contributes to the development of chemoresistance during ovarian cancer progression through amplification of endogenous survival signaling pathways [2], [15], and results presented here suggest that HDAC1 contributes to the loss of RGS10 expression in chemoresistant ovarian cancer cells. To determine HDAC1-mediated changes in cell survival of chemoresistant ovarian cancer cells, cisplatin resistant A2780-AD cells were transfected with HDAC1 siRNA. Following transfection, cells were incubated with 50 µM cisplatin and apoptosis was analyzed using an Annexin V:PE apoptosis detection kit (BD Pharmingen). Annexin V binds phosphatidylserine, which is exposed only in apoptotic cells, while the membrane impermeant DNA label 7-Aminoactinomycin D (7-AAD) selectively binds to GC regions of the DNA only in late apoptotic or dead cells with compromised membranes [30]–[32]. Thus, early apoptotic cells are stained with only annexin V-PE, while late apoptotic and dead cells are stained with both annexin V-PE and 7-AAD. Flow cytometric analysis was used to distinguish between populations of unlabeled and singly- or doubly-labeled cells. HDAC1 knock down significantly increased the population of cisplatin-stimulated cells from 12.9% to 32.1% that are positive for both annexin V-PE and 7-AAD (late apoptotic or dead cells) (Fig. 5A). The results are confirmed by three independent experiments (Fig. 5B). Knock down efficiency of HDAC1 and RGS10 protein expression following the HDAC1 knock down was confirmed in A2780-AD cells by western blot analysis (Fig. 5C). Together these data suggest that HDAC1 mediated reduction of RGS10 expression blunts the ability of cisplatin to induce cell death in A2780/AD ovarian cancer cells.

### DNMT1 knock down decreases HDAC1 binding to the RGS10 promoter in chemoresistant ovarian cancer cells

**Figure 6:**
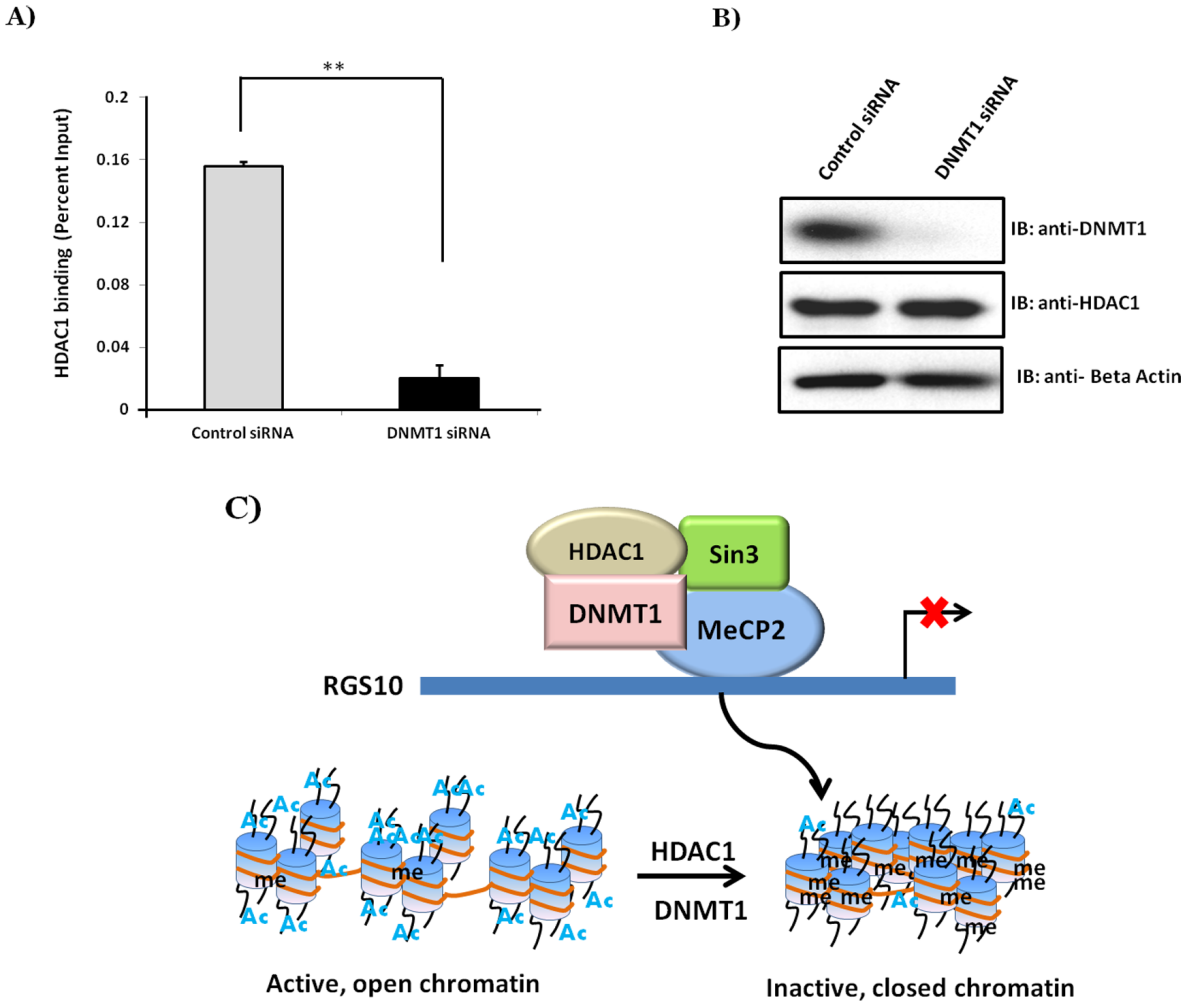
DNMT1 knock down decreases HDAC1 binding to RGS10 promoters in chemoresistant ovarian cancer cells. A2780-AD cells were transfected with HDAC1 siRNA or with control siRNA and were incubated for 72 hours. HDAC1 binding to RGS10 promoters was explored with ChIP assays in HDAC1 siRNA or in control siRNA treated A2780-AD cells. Lysates were immunoprecipitated with control or anti-HDAC1 antibodies. Associated DNA was isolated and analyzed via real time PCR using primers spanning the RGS10 and GAPDH promoters. Real-time PCR values were normalized to the total amount of promoter DNA added (input). Input values represent 5% of the total cell lysate ** p<0.005. **A)** Levels of HDAC1 associated with RGS10 promoters following DNMT1 knock-down in A2780-AD ovarian cancer cells. Data were normalized to GAPDH and values represent mean ± SEM of three independent experiments. **B)** Western blot analysis shows specificity and efficiency of DNMT1 knock down in A2780-AD cells. **C)** Model for mechanism by which RGS10 is suppressed in chemoresistant A2780-AD cells. MeCP2 binds DNA region, containing methylated CpG dinucleotides. MeCP2 directly interacts with DNMT1 to maintain DNA methylation. MeCP2-DNMT1 complex also recruits HDAC1 to RGS10 promoter via binding Sin3 complex.

HDAC1 and DNMT1 contribute to gene silencing through recruiting transcriptional repressors to promoter regions [33]–[35] and work together to suppress gene expression [24], [36]. Our data suggest that HDAC and DNMT activities cooperatively silence RGS10 (Fig. 4C). To investigate crosstalk between these two epigenetic regulators, A2780-AD cells were transfected with DNMT1 siRNA or control siRNA and were incubated for 72 hours. HDAC1 binding to the RGS10 promoter was examined by ChIP assays in DNMT1 siRNA or control siRNA treated A2780-AD cells. DNMT1 knock down significantly decreased binding of HDAC1 to the RGS10 promoter (Fig. 6A) and western blot analysis (Fig. 6B) demonstrated successful and specific knockdown of DNMT1. The converse experiment was also performed where A2780-AD cells were transfected with HDAC1 siRNA. Western blot analysis demonstrated knockdown of HDAC1 resulted in suppression of DNMT1 protein expression (data not shown); an observation seen by others as well [37]. These data suggest that HDAC1 is recruited to the RGS10 promoter via DNMT1 and methyl-CpG binding protein 2 (MeCP2) dependent mechanisms (Fig. 6C). MeCP2 directly interacts with DNMT1 and it is possible a MeCP2-DNMT1 complex recruits HDAC1 to RGS10 promoter via binding Sin3 [38]–[41] or NuRD complexes [23]. In this manner, DNMT1 and HDAC1 synergistically contribute suppression of RGS10 transcription expression as ovarian cancer progresses.

## Discussion

GPCRs have been recognized as important drug targets for treatment of multiple cancers [10], [42], [43]. The strength of signaling pathways initiated by GPCRs is attenuated by members of the RGS protein family, including RGS10 [8], [44]–[46]. DNA methylation and histone deacetylation are often associated with transcriptional repression of gene expression [47], [48] and with decreased responsiveness to chemotherapy [49], [50]. We recently linked the suppression of RGS10 expression to ovarian cancer cell survival and chemoresistance, and further showed RGS10 knock down to increase cell growth and survival [2], [15]. DNA hypermethylation and histone deacetylation contribute to ovarian cancer chemoresistance through amplification of master regulators of multiple cell survival proteins [15], [51]–[53]. Recent findings suggest epigenetic control mechanisms for cisplatin resistance in ovarian cancer, and multiple genes targets that may be subjected to epigenetic control [53]. In our current study, we demonstrate specific contribution of two important epigenetic regulators, HDAC1 and DNMT1, to the suppression of RGS10 expression in chemoresistant ovarian cancer cells.

HDACs remove acetyl groups from substrates, including the histones of chromatin. Conversely, HDAC inhibitors preserve the acetylation status of proteins and induce growth arrest and apoptosis of cancer cells [54]–[56]. Clinical trials show HDAC inhibitors to be effective anti-tumor drugs [57] and HDAC inhibitors have recently shown great therapeutic promise against ovarian cancer [58]–[60]. Our data suggest that reversal of HDAC1-mediated silencing of RGS10 likely contributes to the growth arrest initiated by HDAC inhibitors.

Multiple epigenetic modifications are commonly disrupted during carcinogenesis. Onco-genes undergo hypomethylation on DNA and acetylation and hypermethylation on histones in order to drive enhanced expression [61], [62]. Conversely, DNA hypermethylation and histone deacetylation commonly occur on tumor suppressor genes [63]–[65]. In combination with histone deacetylase inhibition, the addition of a DNA methylation inhibitor has shown robust re-expression of silenced genes in tumor cells [66]. The data presented here further indicate DNMT1 accumulation at the RGS10 promoter suppresses RGS10 transcription in chemoresistant cells.

We previously observed that RGS10 suppressed activation of the survival factor Akt while RGS10 knock down increased activation of Akt; and thus established a causative relationship between suppression of RGS10 and reduced susceptibility to chemotherapeutic cytotoxicity [2]. Thus, RGS10 functions as a tumor suppressor by blunting endogenous survival pathways, with the level of expression of endogenous RGS10 playing a critical role in the determination of apoptosis or survival. Hence, we focused here on therapeutic approaches to decrease chemoresistance by enhancing RGS10 expression in chemoresistant ovarian cancer cells. HDAC1 knock down enhanced cisplatin-stimulated apoptosis in chemoresistant cells, suggesting that knocking down HDAC1 increases RGS10 expression and thus contributes to inhibition of GPCR-stimulated survival signaling pathways, resulting in an increase of apoptotic cells by the chemotherapeutic drug cisplatin.

DNA methylation and histone deacetylation act synergistically to silence cancer-associated genes in ovarian cancer [22]–[24], [67]. Ovarian cancer is associated with elevated expression of DNMTs and HDACs and high-level expression of DNMT1 and HDAC1 is prominent in high-grade ovarian tumors [68]. HDAC1 binds the N-terminus of DNMT1 to form a transcriptional repression complex and the MeCP2 directly interacts with DNMT1 to maintain DNA methylation [69]. To explore interactions between DNMT1 and HDAC1 at RGS10 genes, DNMT1 was knocked down and the level of HDAC1 binding to RGS10 promoters was determined by ChIP. DNMT1 knock down significantly decreased HDAC1 binding to the RGS10 promoter in chemoresistant ovarian cancer cells, indicating HDAC1 is recruited to the RGS10 promoter via DNMT1 and MeCP2 dependent mechanisms. Recent reports suggest DNMT1 and HDAC1 expression increases with ovarian cancer stage [68]. Our results further imply that the increased expression of DNMT1 and HDAC1 results in the generation of repressive complexes which target RGS10 promoters and cooperate in regulating ovarian cancer progression.

The gold standard of care for ovarian cancer is currently a combination of platinum and taxane chemotherapy which, along with second or third line chemotherapy regimens, often does not provide sufficient results [70], [71]. New agents, or new agents in combination with current chemotherapeutic agents, are therefore urgently needed to overcome the drug resistance phenomenon. Gene silencing epigenetic modifications are reversible [72]–[74], thus inhibition of DNA hypermethylation and histone deacetylation can be considered as an adjuvant therapeutic approach for ovarian cancer treatment.

HDAC and DNMT1 inhibitors induce a potent anticancer response by inhibiting histone deacetylation and DNA hypermethylation [75]–[77]. We have shown that inhibition of DNMTs and HDACs by 5-Aza-dC and TSA induced significant RGS10 transcript expression in chemoresistant cells. Of note, the increase in RGS10 transcript expression was similar in HDAC1 and DNMT1 knock down experiments as in experiments with the general inhibitors TSA (HDACs) or 5-Aza-dC (DNMTs), suggesting that HDAC1 and DNMT1 are the primary enzyme subtypes responsible for suppression of RGS10 genes.

Recent reports indicate tumorigenicity and metastasis of ovarian cancer cells is significantly suppressed by the combination of HDAC inhibitor TSA and 5-Aza-dC in xenograft mouse models [78]. Thus, we determined the effects of a combination of TSA and 5-Aza-dC treatments on RGS10 expression and cell viability in chemoresistant ovarian cancer cells. The combination of these two drugs synergistically enhanced RGS10 transcript expression and chemoresistant cell viability. Further, we show that HDAC1 binding to RGS10 promoters in chemoresistant cells is dependent on DNMT activity. A likely mechanism for this suppression of RGS10 is that the DNMT1 repression complex recruits HDAC1 to RGS10 promoters.

A recent phase I clinical trial was the first to attempt to reverse platinum resistance in ovarian cancer with a combination of methylation and histone deacetylase inhibition [72], [79]. These results show that changing methylation and acetylation results in changes in clinical outcomes. Further, preselecting patients based on known methylation status may optimize treatment responses [79]. Our study supports RGS10 genes as targets for demethylating and acetylating therapy and identifies RGS10 de-suppression as a likely contributing mechanism for the clinical efficacy of DNMT1 and HDAC1 inhibitors in the treatment of chemoresistant ovarian cancer.
